# Anneal-Hardening Behavior of Cr-Fe-C Alloy Deposits Prepared in a Cr^3+^-Based Bath with Fe^2+^ Ions

**DOI:** 10.3390/ma10121392

**Published:** 2017-12-05

**Authors:** Ching An Huang, Jhih You Chen, Hai Wang, Po Liang Lai

**Affiliations:** 1 Department of Mechanical Engineering, Chang Gung University, Taoyuan 333, Taiwan; d0322001@cgu.edu.tw; 2 Department of Mechanical Engineering, Ming Chi University of Technology, New Taipei 243, Taiwan; whai@mail.mcut.edu.tw; 3 Bone and Joint Research Center, Chang Gung Memorial Hospital, Taoyuan 333, Taiwan; polianglai@gmail.com

**Keywords:** trivalent Cr electroplating, annealing, microstructure, hardening mechanism

## Abstract

Cr-Fe-C alloy deposits were successfully prepared on high-carbon tool steel in a Cr^3+^-based electroplating bath containing Fe^2+^ ions and suitable complex agents. A Cr-based alloy deposit was obtained with an electroplating current density higher than 25 Adm^−2^, and a Fe-based alloy deposit was obtained using a current density of 20 Adm^−2^. Following electroplating, these alloy deposited specimens were annealed via rapid thermal annealing (RTA) at 500 °C for different periods up to 30 s. The experimental results show that Cr- and Fe-based alloy deposits could be significantly hardened after RTA at 500 °C for a few seconds. The maximum hardness was that of the Cr-Fe-C alloy deposit annealed at 500 °C for 10 s. The maximum hardness of 1205 Hv was detected from the annealed Cr-based alloy deposit prepared with 30 ASD. The hardening mechanism of annealed Cr- and Fe-based alloy deposits is attributed to the precipitation of C-related membranes. The hardness values of the annealed Cr- and Fe-based alloy deposits increase with the increasing degree of crystallization of the C-related membranes.

**Research Highlights:**

1. Cr- and Fe-based deposits were obtained from a Cr^3+^-based plating bath.
2. Cr- and Fe-based deposits can be hardened via RTA at 500 °C for a few seconds.
3. Hardened phase in the annealed alloy deposits is crystalline C-related membranes.
4. Maximum hardness of 1205 Hv was that of the annealed Cr-based deposit.

## 1. Introduction

Because it has superior wear and corrosion resistance, Cr- or Cr-based alloy electroplating is widely used for the surface finishing of metallic components. Generally, Cr or Cr-based alloy deposition can be performed through electroplating from a Cr^6+^-based bath that is highly hazardous for the environment and humans. Therefore, the development of facile Cr-based alloy deposition from a Cr^3+^-based electroplating bath with low toxicity has recently attracted the attention of many researchers [1,2,3,4,5]. Because the hydrated Cr^3+^ ions are stable in solution [4,6], the addition of complex agents such as formic acid and urea in a Cr^3+^-based bath is vital for Cr-based alloy electrodeposition [7,8,9,10,11]. Many studies have confirmed that Cr-C electrodeposition can be achieved from a Cr^3+^-based bath with the addition of formic acid. The C content in the Cr-C deposit is considered to be provided from the formic acid in the Cr^3+^-based plating bath [7,12,13,14,15,16].

Many researchers have proved that the hardness of the as-plated Cr-C deposit can be significantly increased through annealing at 600 °C for 30 min. Based on the results of X-ray diffraction measurements, the hardening mechanism can be attributed to the precipitation of Cr carbides in the Cr substrate [12]. In our previous works [17,18], we proposed that the precipitation of diamond-like membranes in the Cr substrate is the main hardening mechanism for the anneal-hardened Cr-C deposit. Therefore, the as-plated Cr-C deposit could be obviously hardened through flame heating for 1 s or high-frequency induction heating for 0.5 s. In this work, the Cr-C-Fe and Fe-Cr-C alloy deposits were prepared from the Cr^3+^-based electroplating bath containing Fe^2+^ ions and suitable complex agents. The annealing behavior of the as-plated Cr- and Fe-based alloy deposits was studied. Annealing was conducted at 500 °C for different periods up to 30 s using a rapid thermal annealing (RTA) furnace. The microstructures and the hardening mechanisms of the annealed Cr- and Fe-based alloy deposits will be discussed herein.

## 2. Experimental Procedure

The Cr-Fe-C alloy deposits were prepared in a Cr^3+^-based electroplating bath [17] with the addition of 11.15 g/L FeSO_4_·7H_2_O, as well as the complex agents of formic acid, glycine, urea, and a small amount of buffer salts. Electroplating was performed in a three-electrode cell by using a potentiostat/galvanostat (PGSTAT302N, AUTOLAB, Kanaalweg, The Netherlands). A rotating cylinder electrode (RCE) made of pure Cu with a diameter of 9.5 mm and a length of 6.7 mm was used as the working electrode. A platinized Ti-mesh cylinder and an Ag/AgCl electrode in a saturated KCl solution were used as the counter and the reference electrodes, respectively. Prior to electroplating, the Cu-RCE surface was mechanically ground with 2000 grit emery paper, cleaned in ethanol and then acetone, rinsed with deionized water, and finally dried with an air blaster. Electroplating was conducted using current densities of 20, 25 and 30 A/dm^2^ at a temperature of 30 + 1 °C. During the electroplating, the rotational speed of the RCE was kept constant at 300 rpm.

Following the electroplating, the alloy-deposited specimens were heated using rapid thermal annealing (RTA, ULVACRHL-P610CP, ULVAC Technologies, Methuen, MA, USA) with a rate of 3 °C/s from room temperature to 500 °C and annealing at 500 °C for up to 30 s. The hardness value and the standard deviation of an alloy deposit were evaluated with 10 measurements around the middle of its cross section mounted in epoxy. The hardness measurement was performed with a Vickers micro-hardness tester (Micro hardness Tester FM-800e, Future Tech Enterprise, Inc., Holbrook, AZ, USA) using a load of 10 g.

The chemical composition and standard deviation of an alloy deposit were quantitatively determined by 10 measurements along the longitudinal site of the cylinder alloy deposit with an electron probe X-ray microanalyzer (EPMA, JEOL JXA-8200, Japan Electroptics Laboratory Co., Ltd., Tokyo, Japan) with an accelerating voltage of 15 kV and a beam current of 50 nA. The microstructures and the electron diffraction patterns of the as-plated and annealed Cr- and Fe-based alloy deposits were observed using transmission electron microscopy (TEM, JEOL JEM-2100 LaB_6_, JEOL, Ltd., Tokyo, Japan). The focused ion beam (FIB, FEI STRATA FIB 205, Thermo Fisher Scientific, Waltham, MA, USA) was used to mill approximately 100-nm-thick TEM specimens from the cross sections of alloy deposits. The hardening phases in the anneal-hardened alloy deposits were extracted by dissolving the anneal-hardened Cr- and Fe-based alloy deposits in the 50 vol % HNO_3_ + 30 vol % HCl + 20 vol % CH_3_CH_2_OH solution [19]. Finally, a Cu-mesh disc with a diameter of 3 mm was used to take out the membranes or precipitates from the etchant prior to drying in air and in preparation for the TEM study.

The electroplating efficiency of an alloy deposit can be calculated based on the results of its EPMA analysis and weight measurement. Based on the results of EPMA analysis, the weight percentages of Cr and the Fe elements in an alloy deposit could be quantitatively detected. The weight of the alloy deposit was determined from the weight difference of the RCEs before and after electroplating with a precision balance (0.01 mg). The reduction reactions of Cr^3+^and Fe^2+^ ions to Cr and Fe, respectively, are assumed to be the main processes for the alloy electrodeposition. Thus, the total reduction electricity (Q’) for Cr^3+^/Cr, as well as Fe^2+^/Fe in the alloy deposit, can be calculated. The electricity (Q) applied for alloy electroplating is the product of the electroplating current and the period; therefore, the electroplating current efficiency for the alloy electroplating can be calculated by dividing Q’ by Q.

## 3. Results and Discussion

### 3.1. Compositions and Electroplating Efficiencies of Alloy Deposits

**Figure 1:**
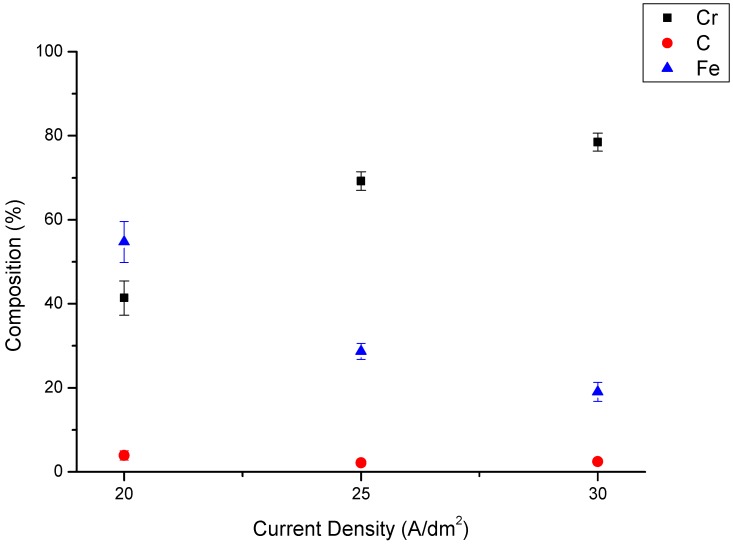
Concentrations of Fe, Cr and C in the Cr-Fe-C alloy deposits prepared in the Cr^3+^-based electroplating bath with Fe^2+^ ions.

Figure 1 shows the Cr, Fe and C concentrations in the Cr-Fe-C alloy deposits prepared with electroplating current densities of 20, 25 and 30 ASD. The examination of Figure 1 shows that the Cr content in a Cr-Fe-C alloy deposit increases with an increasing the electroplating current density; specifically, 41.4 wt % and 78.5 wt % Cr were measured for 20 ASD and 30 ASD, respectively. Conversely, the Fe content in the Cr-Fe-C alloy deposit decreases with increasing electroplating current density, indicating that a Fe-based alloy deposit can be obtained with an electroplating current density lower than 20 ASD, whereas a Cr-based alloy deposit requires 25 or more ASD. Because the Fe^2+^/Fe reduction potential is higher than that of Cr^3+^/Cr, a Fe-based alloy deposit can be obtained at a relatively low cathodic overpotential or electroplating current density regardless of the presence of a small amount of Fe^2+^ ions in the electroplating bath. With a high cathodic overpotential or electroplating current density, a Cr-based alloy deposit can be achieved owing to a relatively high Cr^3+^-content in the electroplating bath. It can be expected that an obvious concentration variation of Fe^2+^ ions could happen adjacent to the cathode during the preparation of a Fe-based alloy deposit because of the low Fe^2+^ concentration in the plating bath. Therefore, the standard deviation values of Cr and Fe contents in the Fe-based alloy deposit are apparently higher than those for the Cr-based alloy deposits (see Figure 1). This is in agreement with our previous study [20], in which compositional segregation was detected in the Cr-Fe-Ni-C alloy deposit prepared in the Cr^3+^-based plating bath containing Fe^2+^ and Ni^2+^ ions. The composition segregation would arise from the depletion of Fe^2+^ and Ni^2+^ ions near the cathode during alloy electroplating.

As shown in Figure 1, the carbon contents of 3.9, 2.1 and 2.5 wt % were detected from alloy deposits electroplated with 20, 25, and 30 ASD, correspondingly, indicating that the C content in the Cr- and Fe-based alloy deposits originated from the complex agents added in the electroplating bath. Based on the results of our previous study [17], we found that for a Cr-C deposit prepared from a Cr^3+^-based electroplating bath, a C-related phase in a membrane shape will be crystallized and the hardness of the Cr-based alloy deposits could be significantly increased after annealing at 600 °C for 1 h. Many crystalline C-related membranes were formed through the transformation of the Cr-based alloy deposit from an amorphous to a crystalline structure during annealing. Moreover, the crystalline C-related membranes could be obtained through reduction-flame heating for 1 s and the high frequency induction heating for 0.2 s [21]. This means that the surface hardening of the Cr-C deposited specimens can be achieved through surface heating for a few seconds.

**Figure 2:**
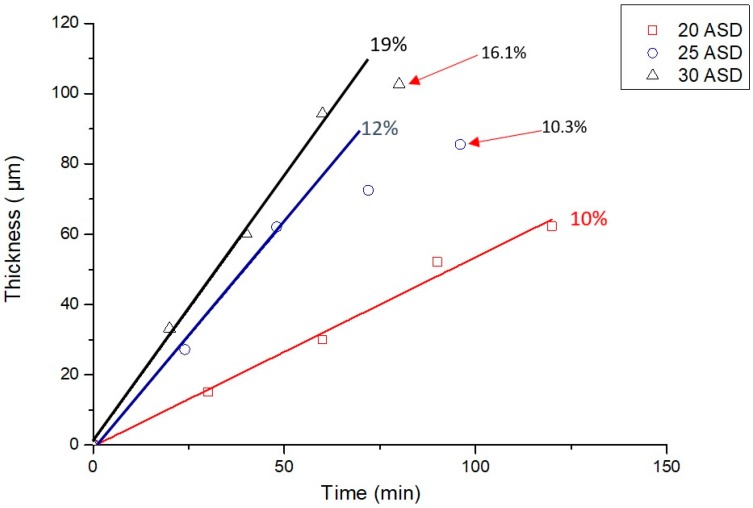
Current efficiencies of Cr-Fe-C alloy electrodeposition in terms of electroplating current densities and periods.

Figure 2 shows the thickness variation and the current efficiencies of Cr- and Fe-based alloy deposits in terms of the electroplating current densities and electroplating periods. The current efficiency of Cr-Fe-C alloy electrodeposition gradually rises with increasing the electroplating current density. The current efficiency of 10% was detected from the electroplating with 20 ASD. It increases to 12% in the electroplating with 25 ASD in the first 50 min, and then gradually decreases to 10.3% after the electroplating for 80 min. The same trend can be found from the electroplating with 30 ASD: its current efficiency is 19% in the first 60 min, and then decreases to 16.1% for 75 min. The decrease in the current efficiency of electroplating with relatively high electroplating current densities could be attributed to concentration depletion of Cr^3+^ and Fe^2+^ ions adjacent to the cathode. That is, the concentrations of Cr^3+^ and Fe^2+^ ions could be obviously consumed near to the cathode with increasing the electroplating time at a relatively high electroplating current density, leading to a decrease in their reduction rates.

Yagi et al. [22] have reported that a Cr-Fe alloy deposit can be prepared using alternating pulsed electrolysis from Cr^3+^-based electroplating bath with a working electrode made of pure Fe. During anodic polarization, abundant Fe^2+^ ions were dissolved close to the Fe electrode surface, and a Cr-Fe alloy deposit could be achieved via the co-reduction of Cr^3+^and Fe^2+^ ions during cathodic polarization. However, a thick Cr-Fe alloy deposit cannot be obtained because of the lack of Fe^2+^ ions after covering the Cr-Fe deposit on the Fe electrode. Although current efficiencies for Fe- and Cr-based alloy deposits are lower than 20%, we confirm that the deposit thicknesses of above 100 μm could be achieved in this study. Based on the results shown in Figure 2, the Cr- and Fe-based alloy deposits with a thickness of approximately 40 μm were prepared for hardness test and microstructure study.

### 3.2. Hardness Test

**Figure 3:**
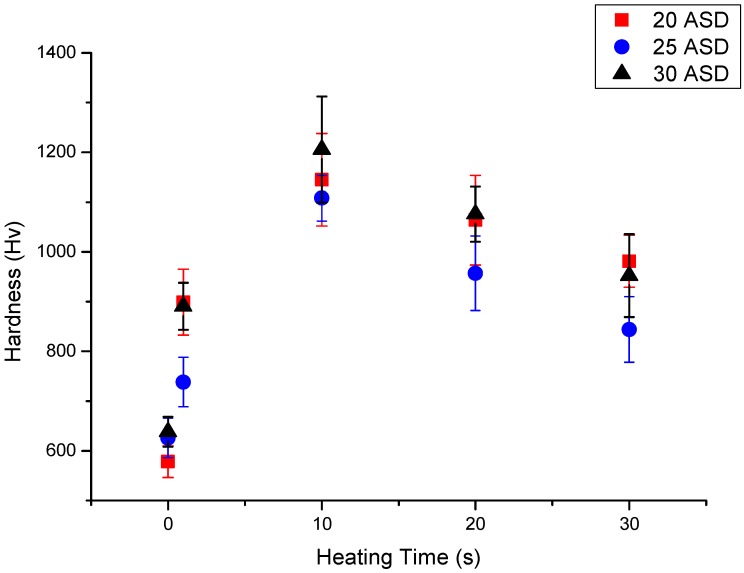
Hardness variation of Cr-Fe-C alloy deposits after rapid thermal annealing (RTA) at 500 °C from 1 to 30 s.

Figure 3 shows the hardness values of the prepared alloy deposits after RTA at 500 °C for up to 30 s. Clearly, the hardness values of the prepared alloy deposits could be obviously increased after RTA at 500 °C for a few seconds. The hardness of a prepared Fe- or Cr-based alloy deposit reaches its highest value after RTA at 500 °C for 10 s, with the hardness gradually decreasing for further annealing. The highest hardness of 1205 Hv was observed for the annealed Cr-based alloy deposit prepared with 30 ASD. The observed hardness for the Cr-based alloy deposit electroplated at 30 ASD is higher than that of the alloy deposit prepared with 20 or 25 ASD after the same annealing. Interestingly, the hardness of the Fe-based alloy deposit prepared with 20 ASD is higher that of the Cr-based alloy deposit prepared with 25 ASD after the same annealing. This suggests that there are differences between the hardening phases of anneal-hardened Cr- and Fe-based alloy deposits.

In our previous study [23], the annealing behavior of Cr- and Ni-rich alloy deposits was investigated after being prepared from the Cr^3+^-based electroplating bath with the addition of Ni^2+^ ions and suitable complex agents. An amorphous Cr-based deposit and a crystalline Ni-based alloy deposit were successfully achieved through electroplating with different current densities. After annealing at 500 °C for different periods, the hardness of the former increased significantly, whereas the hardness of the latter gradually decreased. The increase in the hardness of the annealed Cr-based alloy deposit is attributed to the precipitation of crystalline C-related membranes [18]. On the other hand, the crystalline Ni-based alloy deposit became slightly soft due to the annealing effect. Differing from the annealing behavior of the Ni-based alloy deposit, we confirmed that the Fe-based alloy deposit prepared from a Cr^3+^-based electroplating bath could be significantly hardened after RTA at 500 °C for a few seconds, implying that an amorphous structure could be expected for the as-plated Fe-based alloy deposit.

As shown in Figure 3, the hardness values of Cr- and Fe-based alloy deposits decrease slightly after annealing at 500 °C longer than 10 s. Because the hardening mechanism of an anneal-hardened Cr-based alloy deposit could be the precipitation of hardening phases, the decrease in deposit hardness after annealing longer than 10 s could be attributed to coarsening the hardening phases in the alloy deposit. It must be noted that the hardness values of alloy deposits after annealing for 30 s are still high enough for applications. This implies the surface hardening treatments on the Cr- and Fe-based deposited specimens are feasible. The surface hardening can be accomplished in a few seconds through high-energy concentrated heating methods, such as laser, high-frequency induction and flame heating methods.

### 3.3. Microstructure Study

**Figure 4:**
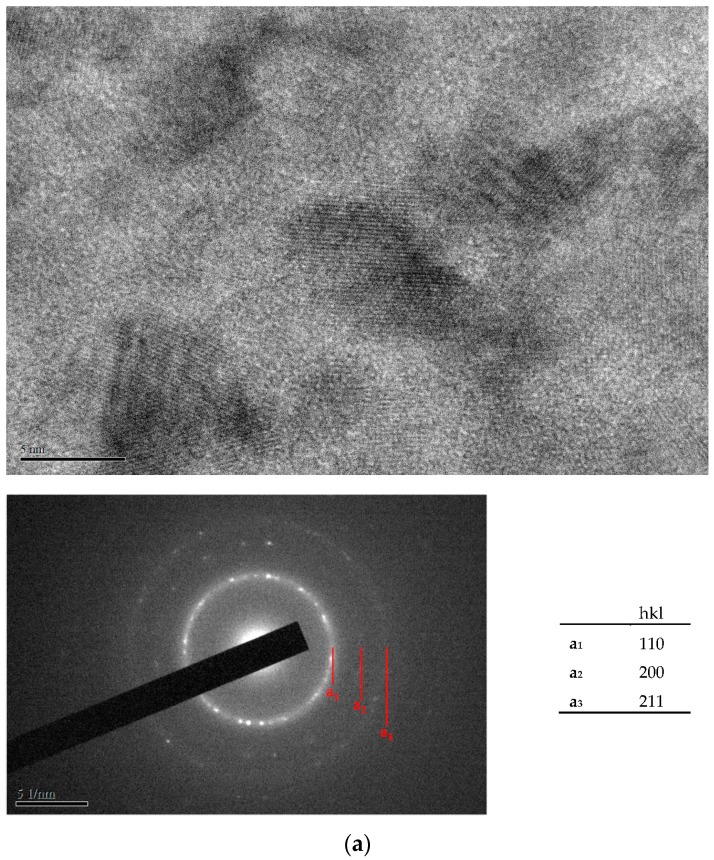

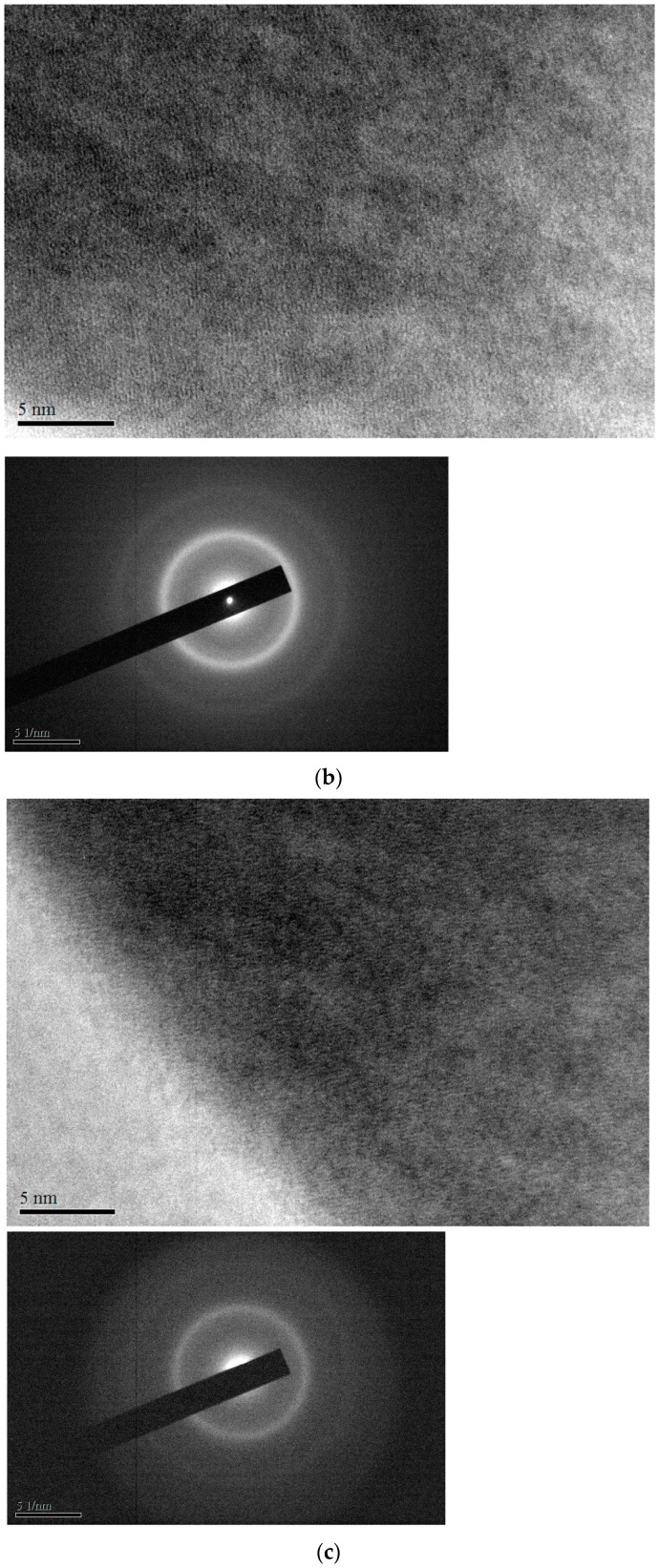
(**a**) Transmission electron microscopy (TEM) micrograph and electron diffraction pattern of as-plated Fe-Cr-C alloy deposit prepared at 20 ASD; (**b**) TEM micrograph and electron diffraction pattern of as-plated Cr-Fe-C alloy deposit prepared at 25 ASD; (**c**) TEM micrograph and electron diffraction pattern of as-plated Cr-Fe-C alloy deposit prepared at 30 ASD.

Figure 4a–c show the lattice images and the electron diffraction patterns of as-plated Fe- and Cr-based alloy deposits prepared with 20, 25 and 30 ASD, respectively. As shown in Figure 4a–c, nearly amorphous structures were observed for the as-plated Fe- and Cr-based alloy deposits. Only a few crystalline particles with a size smaller than 3 nm were found for the as-plated alloy deposits electroplated with current densities of 20 and 25 ASD. A fully amorphous structure was observed from the as-plated Cr-based alloy deposit prepared with 30 ASD. This can be recognized from their lattice images and electron diffraction patterns in which broad faint rings with a halo background were observed.

**Figure 5:**
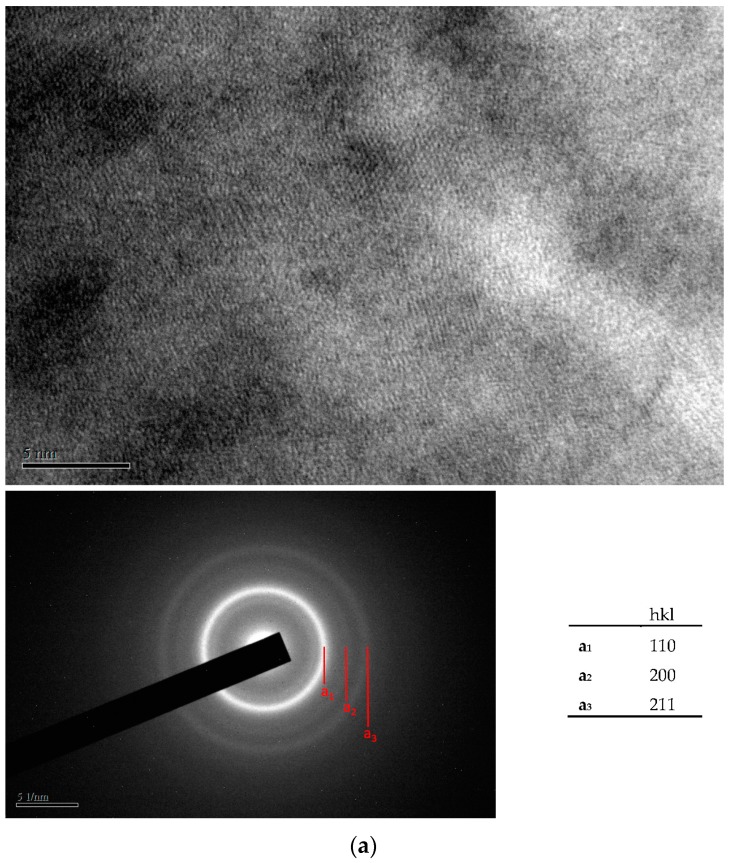

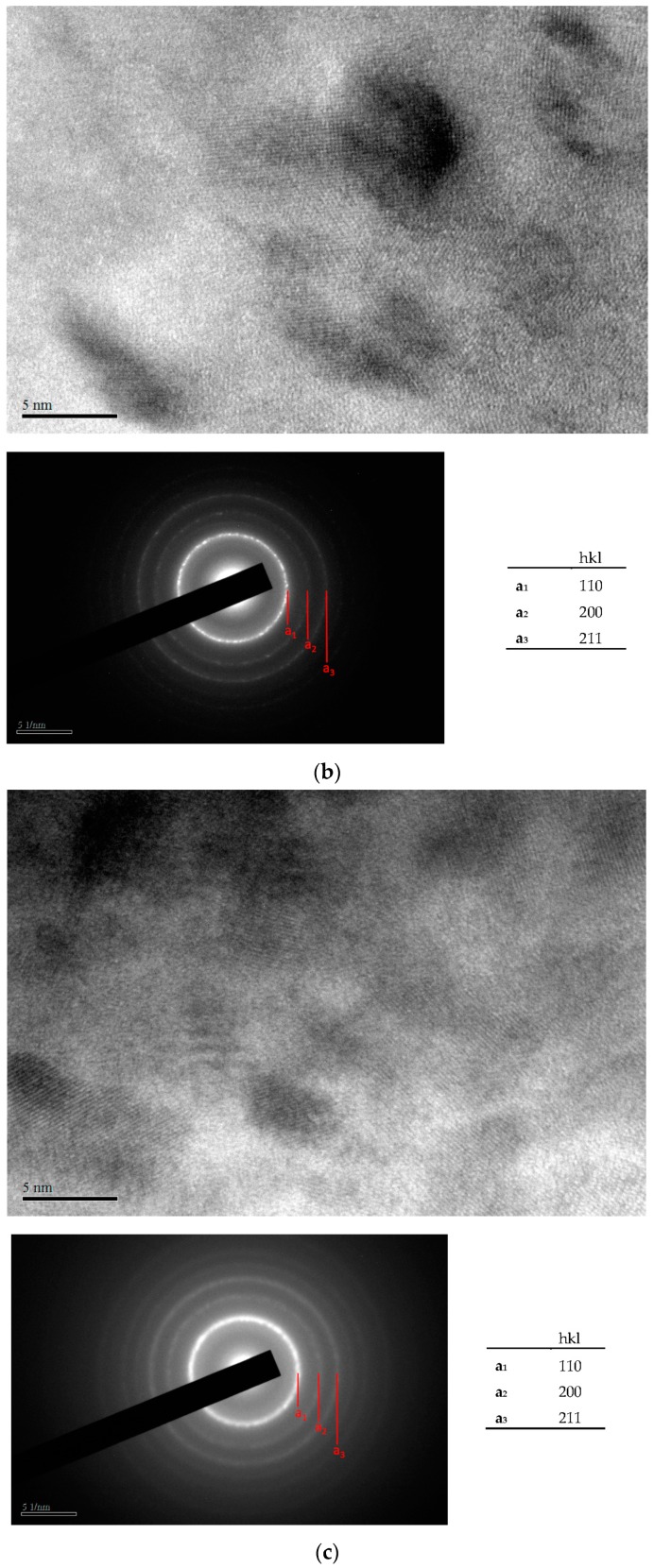
(**a**) TEM micrograph and electron diffraction pattern of anneal-hardened Fe-Cr-C alloy deposit prepared with 20 ASD. The deposit was RTA-treated at 500 °C for 10 s. The diffraced planes a_1_, a_2_ and a_3_ correspond to (110), (200) and (211) planes of Fe with body-centered cubic structure; (**b**) TEM micrograph and electron diffraction pattern of anneal-hardened Fe-Cr-C alloy deposit prepared with 25 ASD. The deposit was RTA-treated at 500 °C for 10 s. The diffraced planes a_1_, a_2_ and a_3_ correspond to (110), (200) and (211) planes of Fe or Cr with body-centered cubic structure; (**c**) TEM micrograph and electron diffraction pattern of anneal-hardened Cr-Fe-C alloy deposit prepared with 30 ASD. The deposit was RTA-treated at 500 °C for 10 s. The diffraced planes a_1_, a_2_ and a_3_ correspond to (110), (200) and (211) planes of Fe or Cr with body-centered cubic structure.

The lattice images of the Fe- and Cr-based alloy deposits after annealing at 500 °C for 10 s are shown in Figure 5a–c; here, a crystalline structure was detected in the anneal-hardened alloy deposits. This can also be deduced from the observed electron diffraction patterns of these deposits in which sharp rings were detected. That is, the prepared alloy deposits markedly crystallized after annealing at 500 °C for 10 s. Grains with a size of 5–15 nm were observed in the annealed alloy deposits (see Figure 5a–c). Many researchers [24] have noted that the precipitation of Cr carbides is the hardening mechanism for the anneal-hardened Cr-C deposit. However, in this study, Cr carbides were not found in the anneal-hardened Cr- and Fe-based alloy deposits. This means that the precipitation of Cr carbides is not the hardening mechanism for the annealed Fe- and Cr-based alloy deposits.

**Figure 6:**
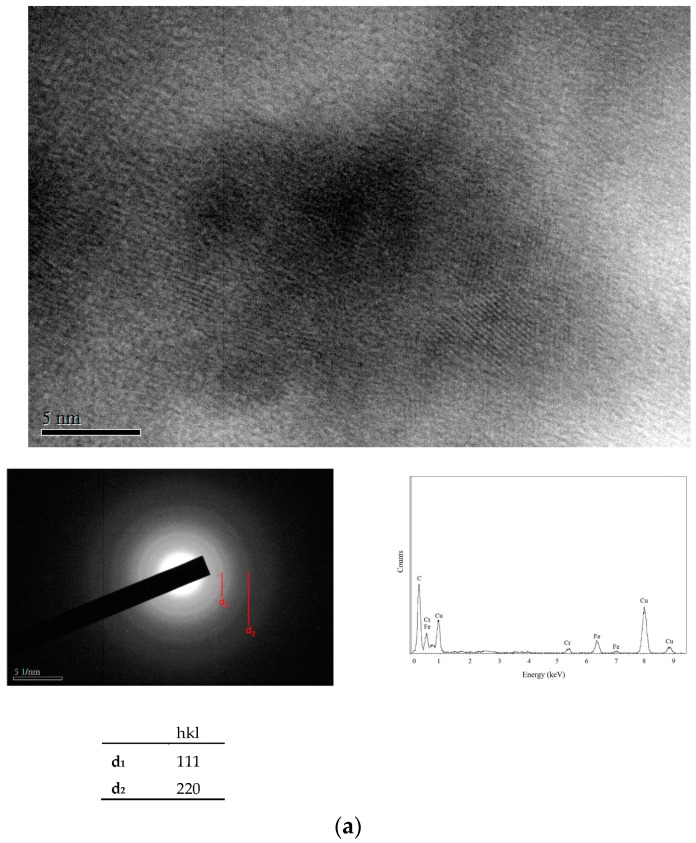

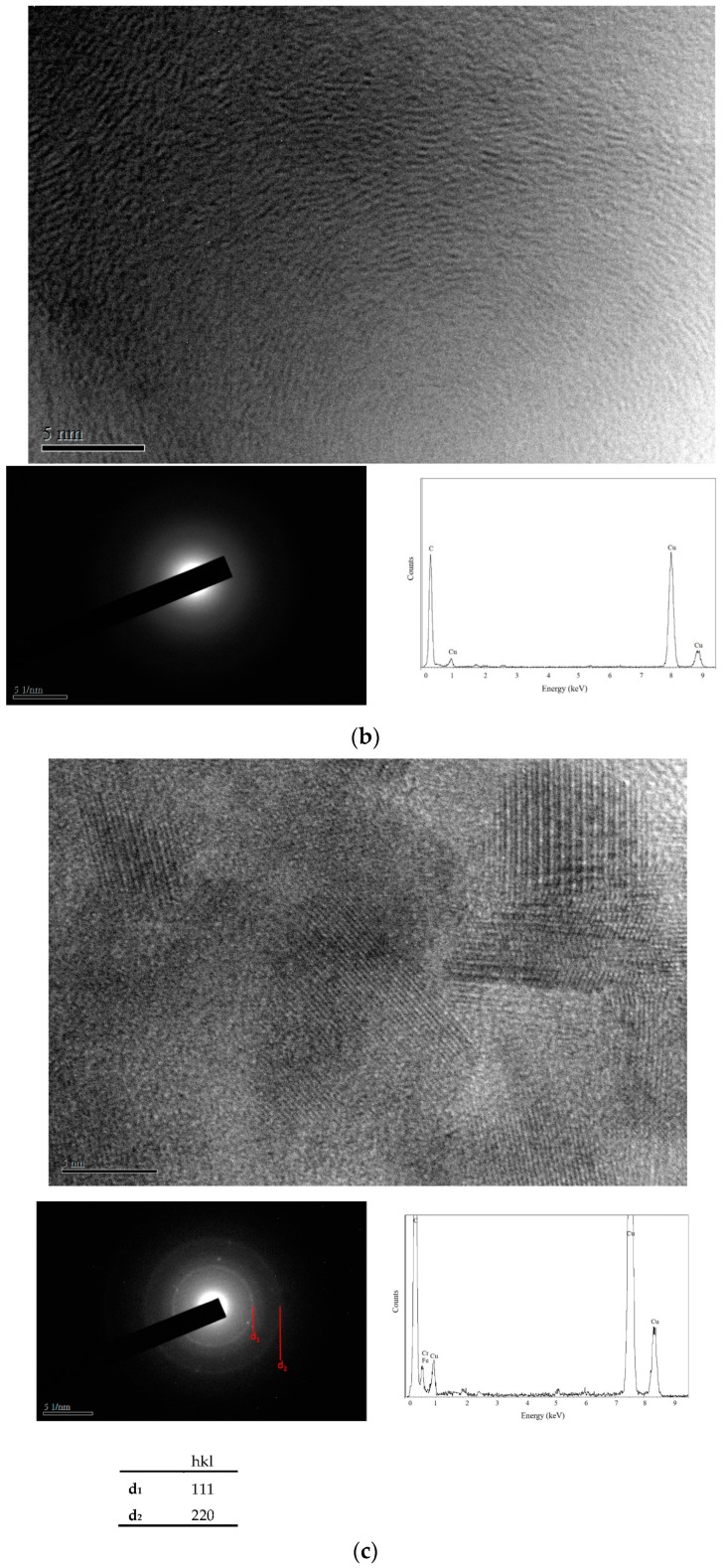
(**a**) Lattice image of the extracted hardening phase in the anneal-hardened Fe-Cr-C alloy deposit prepared with 20 ASD. The deposit was RTA-treated at 500 °C for 10 s. The diffracted plane of d_1_ and d_2_ are corresponding to (111) and (220) planes of the diamond structure; (**b**) Lattice image of the extracted hardening phase in the anneal-hardened Cr-Fe-C alloy deposit prepared with 25 ASD, showing an amorphous structure. The deposit was RTA-treated at 500 °C for 10 s; (**c**) Lattice image of the extracted hardening phase in the anneal-hardened Cr-Fe-C alloy deposits prepared with 30 ASD. The deposit was RTA-treated at 500 °C for 10 s. The diffracted plane of d_1_ and d_2_ are corresponding to (111) and (220) planes of the diamond structure.

The hardening phases of an anneal-hardened alloy deposit were extracted by dissolving the annealed alloy deposit in the 50 vol % HNO_3_ + 30 vol % HCl + 20 vol % ethanol solution, followed by the removal of the hardening phases from the solution. Figure 6a–c show lattice images and EDS (Energy Dispersive Spectroscopy) spectra of the extracted phases from the anneal-hardened Fe- and Cr-based alloy deposits prepared at 20, 25 and 30 ASD, respectively. Similar to the anneal-hardened Cr-C alloy deposit, the extracted phases in a membrane shape are mainly composed of the C element that is believed to be provided from the formic acid complex agent during the electrodeposition [25]. Interestingly, the extracted C-related membranes from the alloy deposits electroplated at 20 and 30 ASD have an obviously crystalline structure, as shown in Figure 6a,c. On the other hand, as shown in Figure 6b, the extracted membrane in the anneal-hardened Cr-based alloy deposit prepared at 25 ASD has a semi-crystalline structure in which the C atoms are arranged in a short range order. The amorphous structure can be recognized from its diffraction pattern shown in Figure 6b in which a round faint background around the transmitted beam was detected. The hardness of the Cr-based alloy deposit prepared at 25 ASD is obviously lower than that of the other alloy deposits after the same RTA treatment. This indicates that the hardness of the annealed Cr- and Fe-based alloy deposits depends on the degree of crystallization of the C-related membranes.

In our previous study [17], some amorphous C-related membranes were detected from as-plated Cr-C deposit prepared from the Cr^3+^-based plating bath. Because the semi- and full-crystalline C-related membranes were found in anneal-hardened Cr- and Fe-based alloy deposits, the amorphous C-related membranes could be developed in the alloy deposits during the reduction of complexed Cr^3+^ and Fe^2+^ ions. Moreover, the C-related membranes transform to semi- or full-crystalline structure after RTA treatment at 500 °C for a few seconds. It can be expected that the volume reduction could take place in an amorphous Cr- and Fe-based alloy deposits by transforming an amorphous to a crystalline structure after annealing at 500 °C for a few seconds. Since the Cr- and Fe-based alloy deposits are well bonded with the steel substrate, they cannot contract freely during and after the RTA treatment at 500 °C. This suggests that a high pressure could be induced in the Cr- and Fe-based alloy deposits when they crystallize from an amorphous structure during RTA treatment. Thus, the C-related membranes tend to crystallize in the RTA-treated Cr- and Fe-based alloy deposits due to the high pressure, as well as the annealing temperature, leading to an obvious increase in hardness of alloy deposits.

## 4. Conclusions

Cr-Fe-C alloy deposits were successfully prepared with a current density varying in the 20–30 ASD range in the Cr^3+^-based electroplating bath with Fe^2+^ ions and suitable complex agents. A Cr-based alloy deposit was obtained with an electroplating current density above 25 ASD, and a Fe-based alloy deposit was obtained for the current density of 20 ASD. Due to the precipitation of crystalline C-related membranes, the Cr- and Fe-based alloy deposits could be significantly hardened after RTA at 500 °C for a few seconds. The hardness values of the annealed Cr- and Fe-based alloy deposits increase with the increasing degree of crystallization of the C-related membranes. The highest hardness of an alloy deposit was observed after RTA at 500 °C for 10 s, and the highest hardness of 1205 Hv was found for the Cr-based alloy deposit prepared with 30 ASD.
